# Reciprocal Trophic Interactions and Transmission of Blood Parasites between Mosquitoes and Frogs

**DOI:** 10.3390/insects3020410

**Published:** 2012-04-03

**Authors:** Laura V. Ferguson, Todd G. Smith

**Affiliations:** Department of Biology, Acadia University, Wolfville, NS B4P 2R6, Canada; E-Mail: 080670f@acadiau.ca

**Keywords:** frogs, insects, parasitism, predation

## Abstract

The relationship between mosquitoes and their amphibian hosts is a unique, reciprocal trophic interaction. Instead of a one-way, predator-prey relationship, there is a cyclical dance of avoidance and attraction. This has prompted spatial and temporal synchrony between organisms, reflected in emergence time of mosquitoes in the spring and choice of habitat for oviposition. Frog-feeding mosquitoes also possess different sensory apparatuses than do their mammal-feeding counterparts. The reciprocal nature of this relationship is exploited by various blood parasites that use mechanical, salivary or trophic transmission to pass from mosquitoes to frogs. It is important to investigate the involvement of mosquitoes, frogs and parasites in this interaction in order to understand the consequences of anthropogenic actions, such as implementing biocontrol efforts against mosquitoes, and to determine potential causes of the global decline of amphibian species.

## 1. Introduction

Mosquitoes (family Culicidae) are well-known pests of mammals due to the anthropophilic nature of a number of species that brings them to our attention. Lesser known are species that preferentially feed on ectothermic hosts such as frogs and snakes. Female mosquitoes require blood in order to produce eggs [1] and in turn, mosquitoes serve as a part of the diet of many species of frogs. Feeding mosquitoes threaten the fitness of their amphibian hosts by harbouring and transmitting parasites [2,3,4] Presence of mosquitoes near or on the body of a frog may promote defensive or avoidance behaviour, including stomping of feet, jumping, and brushing of feet over back and head [5]. The mosquito must avoid this defensive behaviour, as well as death by consumption, while attempting to obtain a blood meal. This interaction between these biting flies and insectivorous frogs thus manifests as a double-edged, predator-prey relationship.

There are three main ways in which blood parasites can be transmitted from an insect vector to a vertebrate host. These include one form of passive transmission in which the parasite is carried by the insect host without engaging in multiplication, and two forms of biological transmission in which the parasite undergoes development or replication in the insect host. Mechanical transmission is characterized by passive transfer of a parasite, specifically on the exterior of the feeding apparatus, into the feeding site on the vertebrate [6]. This type of transmission is not commonly observed with heteroxenous blood parasites of insects and vertebrates, but it is suspected that some arboviruses may be transmitted by both mechanical and salivary transmission [6,7]. 

The most familiar mechanism of biological transmission of blood parasites is by injection of the infectious agent when the insect bites, a method employed most notably by *Plasmodium falciparum* Welch, the causative agent of the deadliest form of human malaria. This salivary transmission involves direct deposition of parasites from the mouthparts of the insect into the feeding site on the vertebrate. Filarial worms [8] and arboviruses [9] are examples of parasites transmitted to frogs by insect vectors during feeding. Trypanosomes of frogs may also be transmitted in the saliva of an insect vector, such as a mosquito; however, the role of mosquitoes in the transmission of trypanosomes is unclear [10]. 

Trophic transmission is another form of biological transmission in which one animal ingests an infected animal. Parasites transmitted trophically from insects other than mosquitoes to frogs include the trematodes *Gorgoderina* spp., *Opithioglyphe* spp., *Ostiolum* spp. and *Halipegus* spp., which typically infect the internal organs such as the bladder, intestines or lungs of the frog following ingestion of infected insects. Egg stages of these parasites are passed through the intestine of the frogs and deposited with faeces. Infective stages of these parasites may be ingested by insects, swept into branchial baskets or may penetrate directly into the tissues of the insect [11]. However, trophic transmission of blood parasites of frogs, such as *Hepatozoon* species, occurs reciprocally through ingestion of infected frog blood by mosquitoes and ingestion of an infected mosquito by frogs [12]. Certain anuran trypanosomes could also be transmitted by this type of reciprocal trophic transmission between mosquitoes and frogs [10], similar to the transmission of avian trypanosomes between birds and black flies [13]. The established reciprocal relationship between frog-feeding mosquitoes and their hosts, as opposed to a one-way predator-prey relationship, is uniquely suited to this form of transmission. Parasites transmitted between these two hosts thus become integrated into the constrained symbiosis that comprises this interaction. 

Mosquito species that feed primarily on ectothermic hosts include *Culex territans* Walker [7], *Culex peccator* Dyar and Knab [14], *Uranotaenia lowii* Theobald [15], *Uranotaenia sappharina* Osten Sacken [14] and *Deinocerites* spp. [16]. *Culex peccator* prefers to feed on reptiles [7], *C. territans*, *U. lowii* and *Deinocerites* spp. prefer amphibian hosts [7,15], and *U. sappharina* may feed equally on both [14]. *Aedes aegypti* Linnaeus are also reported to feed on frogs [17]; however, this species feeds primarily on warm-blooded hosts [18]. *Culex territans* has a wide geographic distribution, including North America, Europe and Africa [19], whereas the remaining species are distributed in southern regions of North America [14], tropical areas of South America [15,16] and the Mediterranean [16]. 

This paper provides an overview of the trophic interactions between mosquitoes, in particular *C. territans*, which is the subject of the majority of studies on the relationships between mosquitoes and frogs, and frogs of the families Ranidae (true frogs) and Hylidae (treefrogs). We highlight the temporal and spatial synchrony of *C. territans* and frogs prompted by this trophic relationship, include an overview of parasites transmitted by mosquitoes to frogs, and discuss in detail the role of the reciprocal trophic relationship in transmission of blood parasites. 

## 2. Habitat Synchrony and Trophic Consequences

### 2.1. Co-Occurrence of Mosquitoes with Different Species of Frogs

Most mosquito species require standing water on or near which to oviposit [20] and may use various freshwater habitats including both seasonal pools [21] and permanent ponds [22] that are also used by frogs for breeding. Many mosquito species prefer seasonal pools in which to oviposit due to lack of fish predators, a characteristic of vernal habitats that is also beneficial to frog tadpoles [21]. Thus, mosquito species, including both those that feed on frogs and those that do not, are commonly found co-occurring with frogs. 

In temperate climates, one of the earliest mosquitoes that overwinter as adults and emerge in the spring is *C. territans.* This species is capable of digesting a blood meal at temperatures of 3.9 °C, compared to a thermal minimum of 10 °C for *Culex pipiens* Linnaeus and *Culiseta melanura* Coquillett. In temperate regions of North America, emergence of *C. territans* corresponds with the emergence of early spring amphibians, specifically wood frogs, *Rana sylvatica* LeConte [22], which are explosive breeders that favour vernal forest pools in which to deposit their eggs. Once wood frogs reach their breeding sites after overwintering, mating occurs quickly, often within a few nights, and oviposition is highly synchronous [23,24]. After breeding is complete, wood frogs retreat from aquatic habitats to moist woodlands [24]. These frogs may serve as the hosts for the first blood meals by *C. territans*; however, *C. territans* may not be stimulated to oviposit in these pools following the first blood meal due to a lack of chemical cues in the water following the rapid exodus of frogs [22]. 

Spring peepers, *Pseudacris crucifer* Wied-Neuwied*,* are small hylid frogs found throughout eastern North America that serve as early spring sources of blood for *C. territans* [18]. Spring peepers emerge around the same time as wood frogs, but may continue breeding for up to two months [25,26]. After breeding, spring peepers move from aquatic sites to woodland habitats during the summer months [26]. Spring peepers may use permanent ponds or ephemeral pools for breeding [27]. 

Continuous breeders in temperate North America include the green frog, *Rana clamitans* Latreille*,* and the bullfrog, *Rana catesbeiana* Shaw, both of which emerge later in the spring and breed from the beginning of June until mid-August in the northern part of their range [28,29]. Both species preferentially inhabit permanent pools of water, such as marshes, swamps, and the shorelines of lakes and slow-moving streams [28]. Green frogs may move away from breeding pools in the late summer in order to forage and build lipid reserves, and may be found in ephemeral pools in forests and other moist terrestrial habitats at this time. However, their spring and summer habitat, and thus host availability to mosquitoes, is consistent.

*Culex territans* larvae are found sympatrically with, and adults of *C. territans* have been recorded to feed on, all four aforementioned species of frogs [5,10,22]. However, the presence of *C. territans* larvae highly correlates only with the presence of green frogs. In the early spring, at temperatures below 9 °C, *C. territans* requires more than 25 d from ingestion of blood to oviposition. If wood frogs were to serve as early blood meals during their breeding season, they most likely will have left the aquatic habitat by the time mosquitoes are ready to oviposit. Thus, vernal habitats used by wood frogs for breeding are generally not suitable for *C. territans* in which to lay eggs since their hosts are not spatially synchronous with an oviposition site [22]. In addition, tadpoles of wood frogs do not metamorphose until July or August, at which time froglets seek out terrestrial habitats [24]. Consequently, suitable hosts would not be available to *C. territans* for a significant portion of the summer. However, wood frogs or spring peepers, including those breeding in permanent bodies of water, likely serve as the source of initial blood meal of overwintering females. For example, the first collection of *C. territans* larvae in New Jersey, USA, in 2004 occurred on May 6, which suggests that overwintering adults took a blood meal at the end of March or beginning of April, concurrent with emergence of wood frogs or spring peepers [22]. Taken together, these observations indicate that oviposition occurs preferentially in permanent water bodies containing green frogs, and not necessarily in the same area where the first blood meal was acquired.

Mosquito species that do not feed on frogs may choose not to oviposit in water that contains tadpoles in order to avoid interspecific competition for overlapping resources that includes bacteria, algae and detritus [30]. Additionally, female mosquitoes may be repelled by chemical cues released in the water by the tadpoles that may signal a threat of predation, similar to detection of kairomones released by mosquitofish, and subsequent avoidance of habitats containing these fish, by ovipositing mosquitoes [31]. Conversely, mosquitoes are attracted to olfactory stimuli released from water that already contains mosquito larvae [20]. It is possible that *C. territans* are attracted to olfactory cues released in water containing green frog tadpoles, despite putative competition between the two for food. As previously mentioned, presence of *C. territans* larvae can be correlated with, and predicted by, the presence of *R. clamitans*. When green frogs shift to vernal habitats in the late summer, *C. territans* larvae appear in these pools approximately two weeks later [22]. There is strong evidence, then, that green frogs are the preferred hosts of *C. territans*, possibly due to the spatial concurrence of an extended site for oviposition in green frog breeding sites and an accessible host for blood meals [22]. The co-occurrence of oviposition sites and preferred frog hosts remains to be elucidated for other species of mosquitoes that feed on frogs.

### 2.2. Trophic Competition in Synchronous Habitats

Use of synchronous habitats by mosquitoes and frogs can lead to competition for resources, as well as predation, and thus trophic relationships exist not only between adult mosquitoes and frogs, but also between larvae of each. Both mosquito larvae and frog tadpoles feed on bacteria, algae and detritus, which may lead to competition for resources. Rubbo and colleagues [21] showed that mosquito survival was not affected by the presence of tadpoles. Conversely, Mokany and Shine [32] and Hagman and Shine [33] observed that, when certain species of mosquito larvae and tadpoles were present together, there was a decrease in the percentage of mosquito larvae that survived, a mean decrease in the wing size or adult body size of those mosquitoes that did survive, and a decrease in tadpole growth rate. Tadpoles were not observed to feed on mosquito larvae [32]. Consumptive competition, as well as mechanical and chemical interferences, may regulate the success of these interacting species, although competition for food may only affect mosquito fitness. Larvae of *Culiseta longiareolata* Macquart also compete with early stage tadpoles of the toad *Bufo viridis* Laurenti for periphyton, and the larval development rate of *Cs. longiareolata* is reduced when *B. viridis* tadpoles are present [34]. Additonally, *B. viridis* tadpoles prey upon early instars of *Cs. longiareolata* [34] and *Hyla septrentionalis* Schlegel actively feeds on larvae of *Culex quinquefasciatus* Say in the laboratory [35]. Interestingly, *Cs. longiareolata* larvae will prey opportunistically on immobile, early stage tadpoles of *B. viridis* [36]. Thus, trophic interactions in the same habitat occur not only between frogs and the adult mosquitoes that feed upon them, but also between tadpoles and mosquito larvae, the adults of which feed on mammals.

Predation of mosquito larvae by aquatic salamanders and their larvae has been observed in the laboratory [37] and there is interest in exploring frogs as natural control of mosquito populations [38]. Mosquitofish have been used in the past to control mosquito larvae, thereby decreasing the number of adult mosquitoes; however, in habitats where mosquito larvae and anuran larvae co-occur, mosquitofish may prey upon frog eggs and tadpoles, effectively decreasing the frog population [38]. Additionally, red-legged frogs, *Rana aurora* Baird and Girard, experience injury as tadpoles and decreased body mass at metamorphosis in habitats containing mosquitofish [39]. Many amphibian populations are in serious decline due to the synergy of several factors, and concern arises that transmission of mosquito-borne human diseases will be exacerbated due to a concomitant increase in mosquito populations [32]. Thus, alternative methods for control of mosquito vectors are needed in concurrence with amphibian conservation efforts. The trophic interactions between mosquitoes and frogs are variable and require further exploration in order to predict future population fluctuations and suitable control measures; for instance, the presence of certain tadpole species negatively affects mosquito control by consuming introduced pathogenic bacteria, effectively negating the application of the biocontrol agent [38].

## 3. Mosquito-Vectored Parasites of Frogs

### 3.1. Trophic Transmission of Hepatozoon Species

Parasites of the genus *Hepatozoon* are apicomplexan blood parasites related to the *Plasmodium* species that cause malaria, and are found in the erythrocytes, and occasionally leukocytes, of terrestrial vertebrates. These protozoa complete sexual development in the gut or haemocoel of various haematophagous arthropods, which must then be ingested by the vertebrate host for transmission to occur. Following asexual development in the liver and other visceral organs of the vertebrate host, intracellular blood parasites are ingested along with a blood meal by the haematophagous arthropod [12].

*Hepatozoon* species are prevalent in many species of true frogs (family Ranidae) and toads (family Bufonidae), and have been reported in several other families. Thus far, the life cycles of only two of 42 species of *Hepatozoon* infecting anurans have been fully elucidated [12,40]. *Hepatozoon clamatae* Stebbins and *Hepatozoon catesbianae* Stebbins are widespread in northeastern North America, and are prevalent in green frogs and bullfrogs, respectively, although both species may be found as mixed infections in each species of frog [40,41]. *Hepatozoon clamatae* is also found naturally in the leopard frog, *Rana pipiens* Schreber. *Culex territans* serves as the definitive host for both species of parasite, which develop as multisporocystic oocysts in the Malpighian tubules [40,42].

The fitness consequences of infection by *Hepatozoon* species are largely unexplored; in snakes, one study suggests that infection is largely benign in most cases [43] but another demonstrates that infection by *Hepatozoon* species may affect growth rate, reproductive output and survival of snakes [44]. Hepatozoonosis of dogs is a fatal disease caused by *Hepatozoon americanum* Vincent-Johnson *et al*. [45] and *Hepatozoon moccasini* Laveran is pathogenic to unnatural lizard hosts [46]. Transmission of *Hepatozoon* species to unnatural hosts, including frog species that do not normally harbour the parasite, could result in more severe fitness consequences. Mosquito fitness during infection by *Hepatozoon* species is largely unexplored; however, mortality has been observed in several species of mosquitoes recently fed on blood containing high levels (5%–10% of erythrocytes infected) of these parasites, including *C. territans* fed on green frogs with moderate infection levels [47]. As well, *C. pipiens* infected with *Hepatozoon sipedon* Smith *et al*., a closely related parasite of snakes, was devoid of eggs during heavy infections of the parasite [48]. Both of these studies suggest that *Hepatozoon* species affect the survivorship and reproductive fitness of their mosquito hosts by as yet undetermined mechanisms. Overall, the effect of *Hepatozoon* species on their hosts is not well known, nor is the effect of any interaction they have in the parasite infracommunity of their frog hosts.

### 3.2. Potential Transmission of Amphibian Trypanosomes by Mosquitoes

*Trypanosoma* species are cosmopolitan flagellate blood parasites of frogs, as well as other vertebrates, and transmission of amphibian trypanosomes is generally considered to occur through the saliva of leeches; however, dipterans may also be responsible for transmission [8,10,17,49]. Trypanosomes may be transmitted to vertebrate hosts through ingestion of a vector, through saliva during blood feeding, or through deposition of faeces by the vector into a wound. All three mechanisms of transmission could occur between an insect vector, such as a mosquito, and a frog [10]. In an experimental setting, *Trypanosoma rotatorium* Mayer will develop in *C. territans* [50]; however, the mosquito apparently does not serve as a natural vector [13]. *Culex territans* has been suggested as a vector for *Trypanosoma ranarum* Lankester [8] and the parasite has been found in wild-caught *C. territans*, regardless if the female was newly engorged or gravid [10], indicating that the parasite survives in the host beyond initial ingestion. Overall, the role of *C. territans* in the transmission of trypanosomes remains ambiguous but potentially relevant.

### 3.3. Salivary Transmission of Filarial Worms

Filarial worms of the genus *Foleyella* are found naturally in the blood of frogs in North America, Europe, Asia and Africa [8,51,52]. Larvae of *Foleyella* species develop throughout the haemocoel but primarily in the fat body of mosquitoes, and they are passed to frogs via salivary transmission when the mosquito feeds [52]. Notably, *C. territans* serves to vector *Foleyella flexicauda* Schacher and Crans among bullfrogs in North America. Microfilariae of these nematodes are ingested when mosquitoes feed on frogs, and later developmental stages are transmitted to frogs when mosquitoes take additional blood meals [53].

*Foleyella* species are reported to cause mortality in frogs harbouring heavy infections of microfilariae or adult worms [4]. Additionally, *Foleyella* species reduce egg production in *Aedes* and *Culex* species [54] and can be lethal to these mosquito hosts, especially when they ingest successive blood meals from an infected frog [18]. The interaction between mosquitoes and frogs during parasite development would thus be interesting to explore to determine whether or not selection favours mosquitoes that do not seek hosts during parasite development. Parasite-induced changes in phenotype are not uncommon [55] and thus may also play a role in regulating the interaction between mosquitoes and frogs.

### 3.4. Mechanical and Salivary Transmission of Viruses

Mechanical and salivary transmission of viruses by mosquitoes may also occur during feeding, which has prompted concern over the possibility of amphibians and reptiles as reservoirs for arboviruses, such as West Nile Virus (WNV) and Eastern Equine Encephalitis Virus (EEEV) that infect humans [7,56]. *Culex territans* tested positive for WNV by the CDC in 2005 [22] and EEEV was detected in pools of *C. territans*, *C. peccator*, and *U. sapphirina* [14,56] although frogs have not been shown to develop detectable viraemia [56]. Frog Erythrocytic Virus (FEV), a virus inside erythrocytes of frogs that may contribute to juvenile mortality, is transmitted to frogs by the bite of a ceratopogonid (biting midge), although *C. territans* may also be responsible for transmission [9]. Additionally, the possibility of frog-feeding mosquitoes as intermediary vectors of Ranavirus, an emerging infectious disease of amphibians, among species of amphibians, or among amphibians and reptiles, such as box turtles, has been suggested [57]. The overall medical and veterinary importance of *C. territans* and other frog-feeding mosquitoes as vectors for arboviruses is not fully elucidated. However, due to the integrated nature of the relationship between *C. territans* and other frog-feeding mosquitoes and their ectothermic hosts, their role in the transmission of such viruses must be considered.

## 4. Host Selection and Preference by Mosquitoes with Consequences for Parasite Transmission

The trophic relationship between mosquitoes and frogs, and any resultant parasite transmission, is dependent on host selection by the mosquito. This involves attraction [58] and repulsion [59] to kairomones, as well as response to visual and auditory cues [58,60]. Mosquitoes that feed on amphibians are highly specialized feeders, and their sensory organs reflect this preference. *Culex territans* has fewer sense organs overall than other *Culex* species, including *C. pipiens* and *C. quinquefasciatus.* Specifically, *C. territans* has fewer blunt type I trichodea, which are considered olfactory receptors that may mediate repulsion to certain odours. They also possess fewer pointed trichodea, known to mediate attractive olfactory activity [61]. Lastly, they possess fewer bulb-shaped trichodea on the maxillary palps, which are associated with CO_2 _detection [62]. This suggests that CO_2_ is not an important attractant for *C. territans* [63] and the overall lower set of olfactory sensilla may indicate very selective odour responses or a reduced dependence on odour-mediated attraction. *Culex territans* must thus rely on other attractant cues in order to find and feed on their amphibian hosts.

Both *C. territans* and *U. lowii* display phonotaxis to amphibian vocalizations [60,15]. Amphibian vocalizations are performed primarily by males during the breeding season, suggesting that mosquitoes are attracted to and may preferentially feed on breeding frogs [7,64]. Feeding peaks for *C. territans* and *C. peccator* coincide with calling peaks of male spring peepers, gray treefrogs (*Hyla versicolor* LeConte) and green treefrogs (*Hyla cinerea* Schneider) [7]. It has also been observed that *C. territans* orients towards amphibian vocalizations played through a CD player. The vocalizations of spring peepers are the most attractive, with the calls of green frogs and bullfrogs as the least attractive, although significantly more attractive than the control sound of a CD player [60]. Using blood meal identification, spring peepers were also determined to be a preferred host for *C. territans* [7]. In contrast, a higher proportion of *C. territans* fed on green frogs (63%) as compared to spring peepers (6%) in a separate study [10]; however, this study was conducted from May until October when green frogs may be more abundant in aquatic habitats than spring peepers. The predictive co-occurrence of *C. territans* with green frogs in permanent ponds [22] may influence the diet of various frog species and consequently the transmission of trophically transmitted parasites such as *Hepatozoon* species.

Attraction to frog calls could also increase the ratio of infected males to females if mosquitoes are more likely to hone in on a calling male during the breeding season. There are no current studies that have been able to draw a link between the sex of frogs and presence of mosquito-borne parasites [15], and indeed one study found that host sex did not have any effect on the density of microfilariae of filarial worms in *Rana vaillanti* Brocchi [65]. However, amphibian trypanosomes, transmitted to green tree frogs, *H. cinerea,* by *Corethrella* species, are found only in sexually mature, male frogs [66]. These midges are also known to be attracted to the vocalizations of male túngara frogs, *Physalaemus pustulosus* Cope, during breeding season [67,68], similar to mosquito species that feed on frogs. Thus, blood parasites transmitted by other biting flies could also display higher prevalence in male frogs. This may occur only in parasites that require salivary transmission, as host-seeking vectors will feed on, and transmit parasites to, vocalizing male frogs. On the other hand, parasites that are transmitted trophically may be ingested equally by both male and female frogs as they feed on infected insects, thereby reducing any bias that sex of the host may have on the distribution of these parasites.

Seasonal shifts in host selection by mosquitoes could affect parasite host specificity; for instance, *Hepatozoon* species are rarely found infecting wood frogs [47]. Although there are several factors that could explain the absence of the parasite, including innate immunity against the parasite or the inability of these parasites to survive when these frogs freeze during the winter, spatial synchrony may also play a role. As mentioned previously, wood frogs are explosive breeders that enter and leave the aquatic breeding site within two weeks. If mosquitoes ingest the first blood meal of the season when the wood frogs reach the breeding site and the parasite requires 30 d to mature in warm laboratory conditions (25 °C), the mosquito may not encounter wood frogs again during the season, thus excluding its suitability as a consistent host for the parasite.

Additionally, abundance of one species does not necessarily sway host preference of mosquitoes. An analysis of blood meals in *C. territans* revealed that they did not feed on cricket frogs (*Acris crepitens* Baird and *Acris gryllis* LeConte), despite the observation that these species were the most abundant at the study site [7]. Mosquitoes did feed on equally small spring peepers, however, suggesting that size did not factor into choice. This suggests that spring peepers are more attractive to mosquitoes, or that a certain characteristic, specifically an auditory or olfactory feature, of cricket frogs may be unattractive to mosquitoes. In some cases, the reciprocal trophic relationship between mosquito and frogs is disrupted by mosquito repellent compounds contained in the skin of certain frog species. *Litoria caerula* White*,* the Australian green tree frog, produces skin secretions that are repellent to both blowflies (family Calliphoridae) and mosquitoes, and two other *Litoria* species repelled mosquitoes as measured by an olfactometer [59]. It is unknown what effects such repellents may have on parasite transmission to these frogs; however, tree frogs of the family Hylidae, including *Litoria* species, have few reported cases of *Hepatozoon* species [12]. This may indicate that certain frog species are more resistant to mosquito-vectored parasites by merit of being unattractive to the vector itself.

## 5. Conclusions

Study of the trophic relationships between frog-feeding mosquitoes and their amphibian hosts provides insight into the evolution of their spatial and temporal synchrony and the life history of parasites transmitted between the two. Parasites, such as *Hepatozoon* species, are interesting to consider in the context of evolution of the trophic relationship between mosquitoes and frogs and the unique synchrony of this reciprocal interaction. The ability of parasites to influence the phenotype of their hosts can shape the interaction of organisms in an ecosystem [69], and the active role of parasites in host relationships must be considered. Parasites that modify the behaviour of their hosts may have the most effect on ecosystem function [2,55] and studies into the behavioural modifications induced by infection are also useful for understanding the relationship between species occupying various trophic levels. Additionally, the interaction between mosquito-vectored parasites of frogs and additional parasites in the infracommunity should also be explored, due to the cumulative and integrative effects that may be imposed on host fitness. 

Studying the relationships between frogs, mosquitoes and their parasites also highlights the integration of trophic levels in an ecosystem. The importance of parasites in ecosystem function is becoming increasingly well recognized [2,55,69] and the loss of parasite species in conjunction with host species decline or eradication is of concern. For example, the current global decline of amphibian species [70] may also result in the coextinction of parasite species infecting frogs, as their hosts disappear. However, host-switching to more abundant hosts may then occur, resulting in parasites infecting new species of hosts with which they held no previous association [71]. Similarly, should frog-feeding mosquitoes be affected by vector control efforts, this could cause the coextinction of mosquito-borne frog parasites, which may predispose frogs to infection with competitor parasites that were previously unable to colonize the host [72], The colonization of novel hosts is often associated with increased virulence [71,72] and thus may negatively affect new species. For example, species of anuran trypanosomes have been documented to be lethal in non-endemic hosts, yet non-pathogenic in their endemic hosts [3]. Thus, we must be aware that even the most resilient of frog species may be at risk as other species of frogs begin to decline.

The trophic relationship of and spatial synchrony between mosquito larvae and frog tadpoles is also an important area of exploration in light of the global decline of amphibian species. For example, it is postulated that frog tadpoles experiencing stress resulting from high densities of insect and fish predators, as well as resource limitation, may be more susceptible to infection with the lethal Ranavirus [73]. Additionally, there is potential for transmission of ranaviruses between sympatric fish and amphibians [74], thus the introduction of fish for the biocontrol of mosquito larvae in areas where frog tadpoles are also present must be carefully monitored. The eradication of mosquito larvae may also reduce available and preferred resources for fish, resulting in higher levels of predation on tadpoles [39]. The consequences of mosquito control may therefore include undesired, negative effects on ecosystem health. In light of the dynamic nature of ecosystems containing frogs, mosquitoes and their parasites during a period of emergence of infectious disease and anthropogenic disturbances, we must be aware of the interconnectedness of species as we move to understand and manage the future of these populations.
